# Medication vs. Movement in ADHD: Interaction Between Medication and Physical Activity on Neurocognitive Functioning

**DOI:** 10.3390/brainsci15101107

**Published:** 2025-10-15

**Authors:** Beverly-Ann Hoy, Michelle Bi, Matthew Lam, Androu Abdalmalak, Barbara Fenesi

**Affiliations:** 1 Faculty of Education, Western University, London, ON N6G 1G7, Canada; bhoy6@uwo.ca (B.-A.H.); mbi5@uwo.ca (M.B.); mlam324@uwo.ca (M.L.); 2 Department of Physiology and Pharmacology, Western University, London, ON N6A 5C1, Canada; aabdalma@uwo.ca

**Keywords:** Attention-Deficit Hyperactivity Disorder (ADHD), physical activity interventions, cognitive and behavioural modulation, functional near-infrared spectroscopy, cortical underarousal in ADHD, children with ADHD, ADHD medication, medication and physical activity, children with ADHD

## Abstract

**Background/Objectives**: Movement during attention-demanding tasks may help compensate for cortical under-arousal in pediatric ADHD patients. However, the influence of medication during movement is unknown. This study assessed the impact of concurrent movement during executive functioning tasks on dorsolateral prefrontal cortex (DLPFC) activation and inhibitory control, with a particular focus on the influence of medication status. **Methods**: Twenty-six children with ADHD (15 medicated; 11 unmedicated) and 24 children without ADHD performed a Stroop task under two conditions: while remaining seated (Stationary condition) and while pedalling on a desk cycle (Movement condition). Functional near-infrared spectroscopy (fNIRS) was used to measure changes in oxygenated and deoxygenated hemoglobin levels in the left DLPFC. **Results**: Sixty-four percent of unmedicated children with ADHD showed greater left DLPFC activity while desk-cycling compared to remaining stationary. Only 37% of medicated children with ADHD showed the same pattern, with 63% showing greater left DLPFC activation when remaining stationary during executive functioning. Children without ADHD had similar DLPFC patterns as unmedicated ADHD children, with 65% showing increased activation during movement. Unmedicated ADHD children who were able to desk-cycle during the Stroop task had higher overall and incongruent accuracy scores; no Stroop differences were found between conditions for children with ADHD who were medicated or for controls. **Conclusions**: Medicated ADHD children did not benefit from physical activity during tasks requiring executive control, yet unmedicated ADHD children showed significantly greater DLPFC activation and inhibitory control when engaging in movement. If medication is not suitable for children with ADHD due to adverse side effects, movement during executive functioning may help mimic the benefit of medications and similarly support attention.

## 1. Introduction

The current frontline treatment recommended to children with ADHD is pharmacotherapy, specifically the use of psychostimulants [1]. These treatments work by targeting catecholaminergic networks in the brain [2], which are responsible for the management and release of essential neurotransmitters such as noradrenaline and dopamine in the prefrontal cortex; these chemicals are critical for executive functioning—core abilities such as inhibitory control, cognitive flexibility, and working memory [3,4], which are essential for everyday tasks and overall well-being. The hallmark symptoms of ADHD include inattention, hyperactivity and impulsivity and are often viewed as tied to a dysfunctional executive functioning system [5,6]. The hypofrontality hypothesis posits that executive dysfunction in ADHD is linked to a decline in blood flow to the prefrontal cortex (PFC) [7]; this diminished blood flow leads to a weakened hemodynamic response, leading to the slower and less effective transportation of oxygen and nutrients needed in activated brain regions [8]. Psychostimulants work in part by upregulating PFC cortical activity, counteracting hypofrontality, improving the hemodynamic response, and promoting executive function [9,10,11]. However, ongoing drawbacks with psychostimulants are their potential for negative side effects—reduced appetite, increased aggression, headaches, gastrointestinal issues, and possible psychosis with long-term use [12]. Furthermore, up to 30% of individuals with ADHD are known as “non-responders” and show no benefit or only adverse side effects to stimulant medications [13].

Physical activity is a well-known behavioural strategy that can help ameliorate ADHD symptoms and supplement alternative treatments, without negative side effects [13,14,15,16]. Physical activity triggers the activation of the same catecholaminergic systems as stimulant medications [17,18] and improves cerebral blood flow throughout the brain and upregulates PFC cortical activity, thereby supporting executive function [9,13,14,19]. Increased gross motor movement in general (i.e., activities that engage major muscle groups throughout the body), which is often labelled as “fidgeting” or “hyperactivity” in those with ADHD, has also been shown to promote PFC activity and executive functioning in children and adolescents with ADHD [9,19]. This suggests an endogenous mechanism that catalyzes the individual to increase their physical movement as a compensatory mechanism for suboptimal cortical blood flow—a natural intelligence that promotes the flow of oxygen and nutrients to areas of focal activity [13,14]. Unfortunately, the predominant Western cultural belief is that fidgeting or hyperactivity is detrimental as it interferes with a child’s ability to focus their attention and learn in traditional sedentary educational contexts [20,21]. However, hyperactivity may be facilitative rather than harmful for executive functioning among those with ADHD, especially around tasks that require focused attention and a sufficiently activated PFC [9,13,14,19]. Recent work has shown that simultaneous movement during attention-demanding tasks promotes PFC cortical blood flow and inhibitory control in children with ADHD [13], especially among those with the hyperactive subtype [14]. This is further supported by work showing how psychostimulants upregulate PFC activity and simultaneously reduce motor activity [9], as the compensatory mechanism of hyperactivity has been addressed pharmacologically. Furthermore, long-term habitual engagement in physical activity may alter and support the brain’s structure and function for optimal cognition and behaviour, along with the physiology underlying ADHD [22,23]. Indeed, regular physical activity has been shown to improve executive functioning and academic achievement among those with and without ADHD [16,23,24]; improve mood, affect, emotional regulation, self-efficacy; and decrease depressive and anxiety-like symptoms in diverse populations [25].

Taken together, psychostimulant therapy and physical activity may be an effective target for ADHD symptoms via overlapping neurocognitive mechanisms. To date, no research has directly compared the effect of stimulant medication on the acute impact of physical activity during executive functioning among children with ADHD. Thus, the current study aimed to address the impact of physical activity on executive functioning in children with ADHD who were either medicated or unmedicated during testing. This work builds on prior research examining key individual difference factors such as ADHD subtype, severity and sex [13,14], and investigates how medication use as another critical individual difference factor impacts the role of physical activity on neurocognitive functioning in ADHD. This study further informs the potential overlapping neurocognitive mechanisms between physical activity and stimulant medication to enhance our understanding of effective interventions for ADHD as well as the disorder’s underlying pathways. We hypothesized that physical activity would support neurocognitive functioning in children with ADHD, whether they were medicated or not, but with a greater magnitude of benefit for those who were unmedicated.

## 2. Materials and Methods

### 2.1. Participants

There were 53 children between the ages of 8 and 12 who were recruited from London, Ontario, through community outreach and institutional clinics. Sample size estimation was carried out with G*Power (version 3.1.9.6). The calculation targeted a medium effect size (f = 0.25), a statistical power of 0.95, and an alpha level of 0.05, focusing on executive functioning as the primary outcome variable within a repeated measures design involving both within- and between-subject factors [13,19,26]. After excluding incomplete data (three participants), the final sample included 26 children diagnosed with ADHD and 23 children without ADHD. Participants with ADHD were further categorized by medication status (15 medicated; 11 unmedicated). Exclusion criteria included limited English literacy, the presence of any developmental or neurological conditions beyond an ADHD diagnosis, physical limitations that prevented moderate-intensity physical activity, and colour blindness.

### 2.2. Measures

#### 2.2.1. Demographic Questionnaire

Table 1 presents demographic information collected from participants and their guardians. Guardians provided information regarding their age, sex, education level, current employment status, and household income. Additional questions focused on the child participants, including their age, sex, ADHD diagnosis, other medical conditions, and current medications.

#### 2.2.2. Diagnostic Screening Using the Vanderbilt ADHD Diagnostic Parent Rating Scale (VADPRS)

Supplementary ADHD-related information, including subtype, was obtained using the VADPRS when necessary. This scale includes the full DSM-V criteria for ADHD and consists of 18 items addressing symptoms of inattention and hyperactivity/impulsivity. Each behaviour is rated by guardians on a 4-point scale, with 0 indicating ‘never’ and 3 indicating ‘very often’. Ratings on the VADPRS using the 4-point scale (“never” to “very often”) denoted behaviour severity. A total score was calculated using the 4-point scale using questions 1–18, which targeted inattentive- and hyperactive/impulsive-specific symptoms. The total score was computed into a percentage and sorted from lowest to highest to demarcate the range of severity and categorized according to low severity (31–49%), moderate severity (50–67%), and high severity (68–90%). VADPRS data are presented in Table 1.

#### 2.2.3. Executive Functioning: Stroop Task

**Figure 1:**
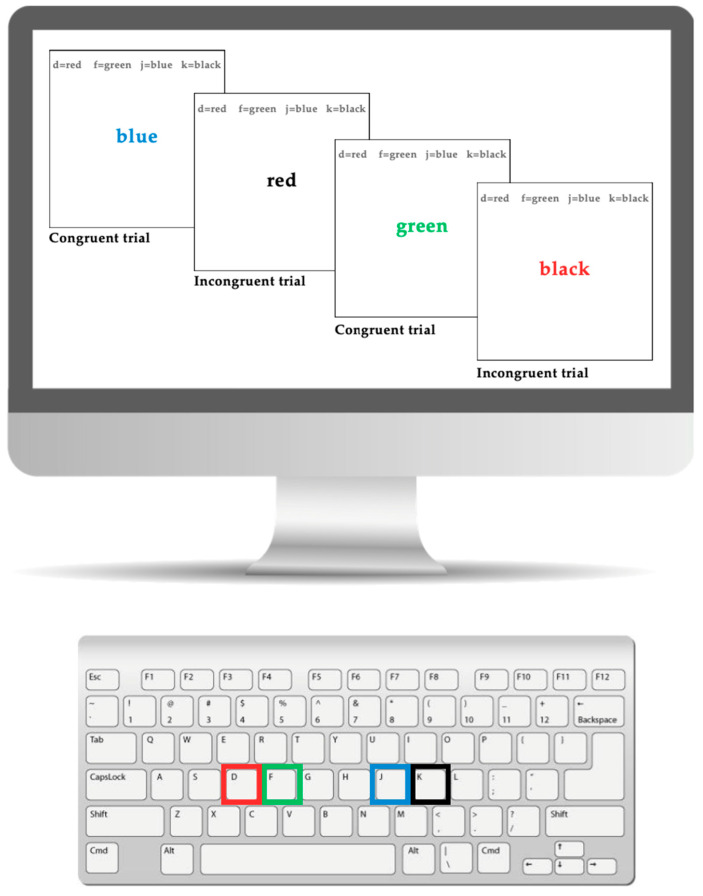
The Stroop task displayed on a computer and keyboard.

The Stroop task serves as a reliable measure of inhibitory control in children with ADHD [27,28]. A computer-based Stroop task (illustrated in Figure 1), designed using the Inquisit platform, was administered to assess inhibitory control. Participants responded to colour-word stimuli, where the text either matched (congruent) or conflicted (incongruent) with the ink colour. Each response key (“D”, “F”, “J”, “K”) on the keyboard was linked to a specific colour (red, green, blue, black). For example, in a congruent trial, the word “red” may appear in red ink, prompting the participant to press the “D” key. In contrast, during an incongruent trial, the word “red” might be displayed in blue ink, creating a conflict between the word’s meaning and its colour; the correct response would be the “J” key for blue. Participants completed four 50 s task blocks separated by 20 s breaks. Both reaction time and accuracy were recorded.

#### 2.2.4. Heart Rate

Continuous heart rate data, collected via an Apple Watch and the VeryFit 2.0 app, was used to gauge the intensity of desk-cycling. The VeryFit app was set to “Workout” mode to track physical activity. Throughout each condition, the Apple Watch continuously sent heart rate data to the app, and the average for each condition was recorded.

#### 2.2.5. Functional Near-Infrared Spectroscopy (fNIRS): Left DLPFC

Prior studies have implicated the left dorsolateral prefrontal cortex (DLPFC) in mediating the impact of physical activity on cognitive control, as measured by performance on the Stroop task [27,29]. Based on this evidence, the left DLPFC served as the main region of interest (ROI) for the current analysis. To monitor neural activity in this region, functional near-infrared spectroscopy (fNIRS) was employed, a non-invasive and portable brain imaging method often used in pediatric research [28]. The NIRSport system was chosen for this study due to its strong suitability to meet the needs of this research [27]. Brain activation triggers increased oxygen demand, leading to elevated blood flow to the active region—a process commonly described as neurovascular coupling [28,30]. FNIRS tracks changes in oxygen delivery and usage by measuring concentrations of oxygenated hemoglobin (HbO) and deoxygenated hemoglobin (HbR), which serve as indirect markers of neural activity [30].

The fNIRS cap used in this study (NIRSport 2, NIRx Medical Technologies, LLC, Minneapolis, MN, USA) was configured with an 18-channel system designed to monitor activity across the prefrontal cortex, operating at a sampling rate of 10.2 Hz. Data acquisition and montage setup were carried out using Aurora software by NIRx (2021.4). To ensure data quality, signal integrity was evaluated using both the coefficient of variation (CV) and signal level metrics, complying with NIRx recommendations that required <3% CV values and signal levels equal to or greater than 3 mV. The cap layout consisted of eight light sources emitting dual wavelengths (760 and 850 nm) and eight detectors, with a minimum spacing of 3 cm between them.

During the experiment, the fNIRS cap was carefully fitted to each participant’s head, ensuring that the infrared optodes were properly aligned on the scalp. Near-infrared light from the optodes traversed the scalp and skull, with part of the light being absorbed by brain tissue. The remaining light was detected by nearby sensors, allowing researchers to assess how much light was absorbed. The data collected allowed for the assessment of dynamic changes in concentrations of oxygenated hemoglobin (HbO) as well as deoxygenated hemoglobin (HbR). Calculations were based on the modified Beer–Lambert Law, a principle that models the variation in light intensity between the point of emission and the point of detection [28].

### 2.3. Design

Participants underwent both the movement and stationary conditions, as part of a within-subjects design, with the order of conditions randomized to control for order effects.

#### 2.3.1. Movement Condition

**Figure 2:**
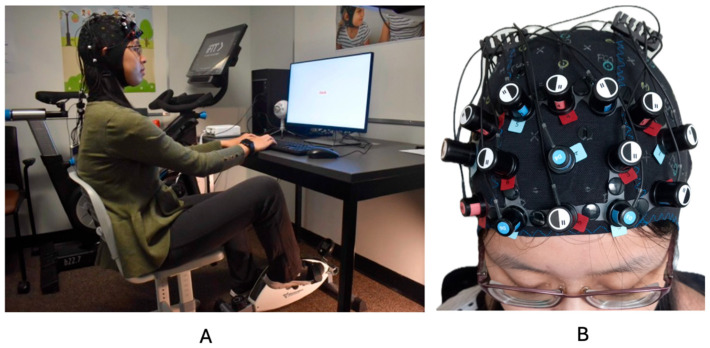
(**A**) Displays the full experimental setup; (**B**) displays the fNIRS cap configuration. This image is reproduced from previously published work by Hoy et al., 2024 [13].

To incorporate physical activity during the Stroop task, participants used a 3D Innovations Desk-Cycle placed beneath the desk [6,31]. Prior to each session, the setup was customized for individual comfort–this involved modifying the chair height, the spacing between the participant and the desk or keyboard, and the positioning of the pedals. Pedalling resistance was configured to the lowest level, and participants were instructed to cycle at a pace they found comfortable and sustainable throughout the session. The average heart rate recorded during this condition was 93.12 bpm (SD = 8.48), confirming light aerobic activity. Figure 2 provides a visual representation of the experimental configuration.

#### 2.3.2. Stationary Condition

During the stationary condition, participants maintained a seated position with their feet resting flat on the floor while completing the Stroop task and undergoing fNIRS recording. The average heart rate during this condition was 85.56 (SD = 8.49).

### 2.4. Procedure

The study took place at Western University in the Faculty of Education. If feasible, participants with ADHD were requested to withhold their medication for 24 h prior to the session; 11 complied by abstaining, while 15 remained on their medication during testing. After obtaining agreement from participants and their guardians to participate, researchers collected consent and assent forms, as well as the VADPRS and demographic questionnaire. The participant was then brought to a nearby room and was introduced to the experimental procedures. To prepare for fNIRS recording, the participant’s head circumference was measured using a fabric measuring tape to identify the suitable cap size. The participant was invited to handle the fNIRS cap and feel the infrared optodes to help ease any apprehension. Participants were instructed to remain motionless for an estimated duration of 30 s while the researchers calibrated the fNIRS system, optimizing the signal quality by ensuring the coefficient of variation (CV) remained under 3%, with signal levels equal to or exceeding 3 mV. After calibration, participants were shown a live display of their brain activity and were given a chance to ask additional questions.

**Figure 3:**
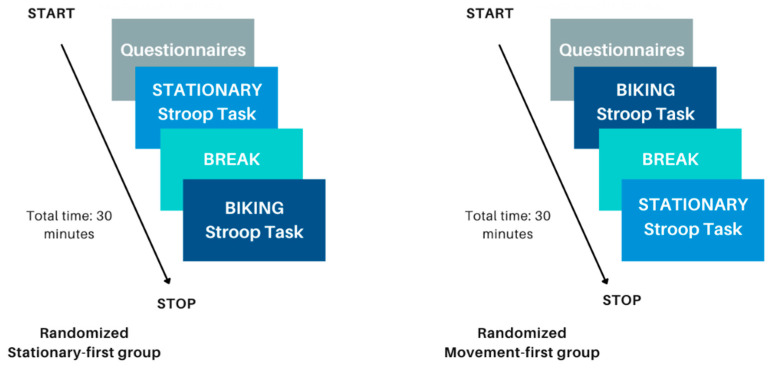
This figure depicts the step-by-step flow of the experimental procedure. The left side illustrates the procedure for participants assigned to the stationary condition first, whereas the right side represents those who began with the movement condition. This image is reproduced from previously published work by Hoy et al., 2024 [13].

Figure 3 illustrates the study procedure. Participants were first given instructions on how to perform the Stroop task and completed approximately three to five practice trials to familiarize themselves before starting the actual task. Each participant completed both the stationary and movement sessions, with order randomized. Before starting the movement condition, participants cycled for one minute to reach a consistent pace. In both conditions, the Stroop task was divided into four 50 s blocks with 20 s breaks. A debrief followed the final condition, during which remaining questions were addressed.

### 2.5. Statistical Analysis

#### 2.5.1. fNIRS and Left DLPFC Activity

The current study utilized data sourced from a larger study previously published in *Brain Sciences* [13]. Preprocessing and analysis of the fNIRS data were carried out using Satori software (version 2.06). Raw data were first trimmed by removing 10 s preceding the initial trigger and 10 s following the final trigger. The resulting segments were then converted to optical density values and underwent motion artifact correction through spike removal and Temporal Derivative Distribution Repair (TDDR) methods [31]. Using the modified Beer–Lambert law, optical density data were converted to reflect changes in hemoglobin concentration. To minimize noise, the data were band-pass filtered within a frequency range of 0.01 to 0.5 Hz. Following normalization, individual general linear model (GLM) analyses were performed to examine differences in neural activity between the movement and stationary conditions for each participant. The left DLPFC was mapped in the fNIRS setup using channels corresponding to the F1, F3, and F5 locations (specifically, channels S4-D2, S1-D2, and S1-D1, respectively). Paired *t*-tests were employed to evaluate differences in oxygenated (HbO) and deoxygenated hemoglobin (HbR) levels within these ROI channels across conditions. Activation of the region was determined if at least one channel showed a statistically significant effect. One participant was removed from the analysis due to poor signal quality.

#### 2.5.2. Executive Functioning

Statistical analyses of Stroop task performance were conducted using SPSS version 30. Due to a data collection error, one participant from the ADHD group was excluded from the final dataset, resulting in a Stroop analysis sample of 25 ADHD participants (15 medicated and 11 unmedicated). A 2 (condition: movement vs. stationary) × 3 (group: medicated ADHD, unmedicated ADHD, control) mixed factorial ANOVA was conducted for key outcome variables: total reaction time (RT), total accuracy (proportion correct), and both RT and accuracy within congruent and incongruent trials. To further investigate within-group patterns, exploratory paired-sample *t*-tests were conducted separately for each group.

## 3. Results

To examine whether counterbalancing the order of movement and stationary conditions influenced the results, six 2 × 2 mixed factorial ANOVAs were performed on Congruent and Incongruent Stroop RT and proportion correct scores. The analyses included condition order (stationary-first vs. movement-first) alongside the primary factors. Findings revealed no significant impact of counterbalancing on any EF measures (*F*s < 3.49, *p*s > 0.07, all ηp^2^ < 0.07), suggesting that the order of conditions did not affect the experimental manipulation’s outcomes. There were no extreme outliers across analyses (SPSS step of 1.5 × interquartile range). Heart rate values were consistently higher during the movement condition compared to the stationary condition in all groups, all *t*s > −4.8, all *p*s < 0.004, all *d*s = 0.87−1.15.

### 3.1. fNIRS and Left DLPFC Activity

**Figure 4:**
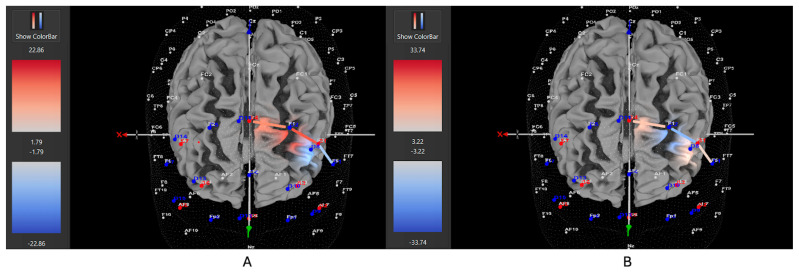
These images represent an unmedicated participant with ADHD, showing oxygenated hemoglobin concentrations in the left DLPFC under the (**A**) movement and (**B**) stationary conditions. The red shading highlights increased oxygenated hemoglobin in the regions of interest (F1, F3, F5) corresponding to the left DLPFC during the (**A**) movement condition. The visuals are filtered to display only the channels located over the ROI.

**Figure 5:**
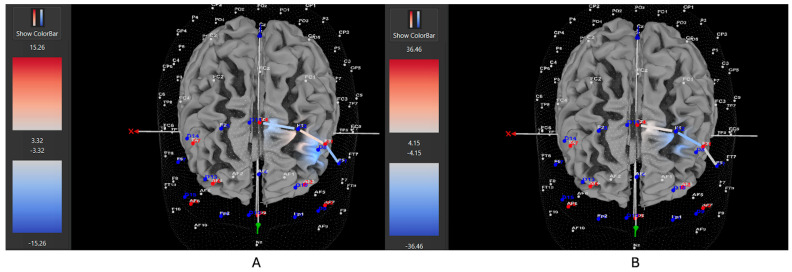
These images represent a medicated participant with ADHD, showing oxygenated hemoglobin concentrations in the left DLPFC under the (**A**) movement and (**B**) stationary conditions. The blue shade indicates reduced oxygenated hemoglobin in the regions of interest (i.e., F1, F3 and F5) corresponding to the left DLPFC, with similar patterns observed across both conditions. The visuals are filtered to display only the channels located over the ROI.

**Figure 6:**
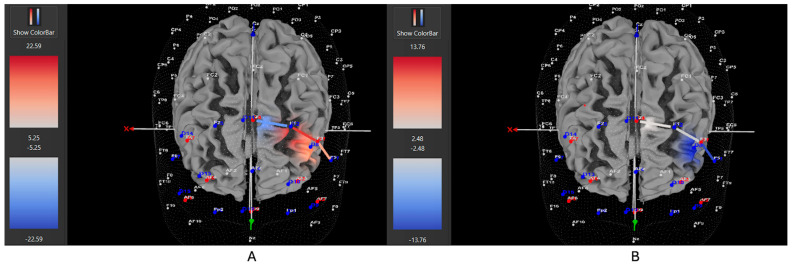
These images represent a participant without ADHD (Control), showing oxygenated hemoglobin concentrations in the left DLPFC under the (**A**) movement and (**B**) stationary conditions. The red shading highlights increased oxygenated hemoglobin in the regions of interest (F1, F3, F5) corresponding to the left DLPFC during the (**A**) movement condition. The visuals are filtered to display only the channels located over the ROI.

Among ADHD participants who were unmedicated during testing, 7 of 11 participants (64%) exhibited increased HbO levels in the left DLPFC during the Movement condition (all βs −0.38–0.88, all *t*s > 2.02, all *p*s < 0.04), while 4 out of 11 (36%) showed higher HbO in the left DLPFC during the Stationary condition compared movement (all βs 0.35–1.48, all *t*s < −2.86, all *p*s < 0.04). Among ADHD participants who were medicated during testing, only 7 of 15 participants (47%) demonstrated higher HbO levels in the left DLPFC during the Movement condition (all βs −0.34–0.93, all *t*s > 5.40, all *p*s < 0.001), while 8 out of 15 (53%) exhibited greater HbO during the Stationary condition (all βs −1.44–0.91, all *t*s < −3.25, all *p*s < 0.001). Among Control participants, 15 of 24 participants (63%) revealed higher HbO levels in the left DLPFC during the Movement condition compared to the Stationary condition (all βs −0.86–1.41, all *t*s > 2.16, all *p*s < 0.03). Conversely, 9 out of 24 (37%) showed greater HbO during the Stationary condition in the left DLPFC (all βs −1.14–0.79, all *t*s < −2.5, all *p*s < 0.01). Figure 4, Figure 5 and Figure 6 display example images of HbO concentration changes during the Stroop task for unmedicated ADHD participants, medicated ADHD participants, and Control participants, respectively, comparing Movement and Stationary conditions. A detailed breakdown of the fNIRS t-test findings can be found in Supplementary Table S1.

### 3.2. Executive Functioning: Stroop Task

Table 2 presents EF descriptive statistics for both the ADHD and the Control groups. For all mixed factorial ANOVAs, there was no main effect of condition (all *F*s < 3.651, all *t*s > 0.062, all ηp^2^ < 0.074), no main effect of group (all *F*s < 1.253, all *t*s > 0.295, all ηp^2^ < 0.052), and no interaction (all *F*s < 2.082, all *t*s > 0.136, all ηp^2^ < 0.083). Exploratory paired t-test analyses within the medicated ADHD group and the Control group yielded no differences in Stroop outcomes between the movement and stationary conditions (all *t*s < 1.394, all *p*s > 0.093, all *d*s < 0.514). However, for the unmedicated ADHD group, they had higher total Stroop and incongruent Stroop proportion correct outcomes when engaging in movement rather than remaining stationary (total Stroop proportion correct, *t*(10) = −1.842, *p* = 0.048, *d* = 0.757; incongruent Stroop proportion correct, *t*(10) = −2.257, *p* = 0.024, *d* = 0.909). All other Stroop outcomes were not significant (all *t*s < 0.983, all *p*s > 0.175, all *d*s < 0.364).

## 4. Discussion

The current study evaluated how movement affects DLPFC activation and inhibitory control during executive functioning in children with ADHD who were either medicated or unmedicated during testing. Interestingly, children who were medicated did not benefit from movement during executive functioning, whereas children who were unmedicated revealed greater activation in the DLPFC and better performance on the Stroop task. These results suggest that if psychostimulant medication is not suitable for children with ADHD due to adverse side effects, movement during attention-demanding tasks may help mimic the benefit of medications and support focused attention in a similar way.

Many children with ADHD cannot tolerate psychostimulant medications due to their negative side effects [2,12]. This leaves many families looking to alternative forms of therapy to manage the myriad of symptoms associated with the disorder, including executive dysfunction, sleep disturbances and mood-related disorders [32,33,34]. Engaging in regular as well as concentrated bouts of physical activity has been repeatedly shown to benefit children with ADHD and improve executive functioning skills [16], academic achievement [16], mood and overall well-being [16,17,22,35,36]. More recently, work has shown that children with ADHD also benefit from simultaneous movement during attention-demanding tasks [9,13,14,19], showing improved DLPFC activation, inhibitory control, and self-efficacy. In the current study, engaging in movement simultaneously during the Stroop task elicited increased DLPFC activity and inhibitory control in children with ADHD, specifically for those who were unmedicated during testing. This finding aligns with previous work showing physical movement activates the same catecholaminergic and arousal networks as stimulant medications [17,18], working to increase cortical blood flow and bring along essential neural resources to areas of focal activity [9,13,14,19,27]. This is especially true for those without the support of psychostimulants who require alternative forms of support to upregulate cortical hypoarousal and support cognitive processing. Other suggested alternatives to psychostimulants are behaviour management interventions such as cognitive behaviour therapy [37], executive function training interventions such as working memory training, attention training and neurofeedback [38], psychoeducation [1,38], transcranial current stimulation, mind–body interventions such as meditation and mindfulness [39], with some research suggesting dietary interventions can also mitigate ADHD symptoms [40,41].

Children with ADHD who were on psychostimulant medication during testing did not benefit from movement during the executive functioning task. This finding is in alignment with previous work showing that psychostimulants are associated with an increase in cerebral blood flow and improved executive functioning [9,10,11]. Additionally, psychostimulants are associated with decreased gross motor activity, i.e., less hyperactivity [19]. Thus, the need for an alternative mechanism, such as physical movement, to upregulate critical processes to support focused attention is diminished due to the efficacy of psychostimulants on the central nervous system [10,11,19]. As a result, children with ADHD who remained on psychostimulants during testing did not require movement to facilitate focused attention. These findings also further support the notion that medication and movement share overlapping neurocognitive mechanisms, and rather than acting in an additive way when combined, one approach may negate the need for the other. Nonetheless, it is critical to emphasize that these findings do not undermine the importance of participating in frequent physical activity for children with ADHD, even if medicated, as the long-term structural and functional processes supported by physical activity extend far beyond the acute effects of movement on the brain [31,41]. Furthermore, psychostimulant medications, even if effective in treating executive dysfunction, are very often associated with disturbed circadian rhythms and sleep [42,43], further exacerbating symptoms in highly comorbid mental health disorders such as depression and anxiety [32,33,34]. Thus, while movement during executive functioning may not be required to support immediate neural activation and performance, the sustained benefits of physical movement for the ADHD brain are undeniable.

It is also important to note that alternative frameworks of ADHD look beyond arousal dysregulation as the mechanistic underpinnings of the disorder. The Cognitive Energetic Model (CEM) underscores the need to account for both cognitive and physiological influences when evaluating and managing ADHD [44,45]. Specifically, the CEM posits that executive dysfunction in ADHD may arise from inadequate energetic states instead of solely from neurocognitive impairments. For example, those with ADHD may function similarly to their neurotypical peers in adequately enriching and stimulating environments but tend to have difficulty in dull or repetitive situations due to insufficient neural activation. Classrooms are traditionally low-stimulation, repetitive settings, where many of the inattentive and hyperactive symptoms of ADHD emerge [46]. The CEM offers a dynamic and context-sensitive perspective beyond a purely structural model of brain function.

### 4.1. Practical Implications

Classroom environments are the most cited places where ADHD symptoms emerge. As previously discussed, this is likely due to their repetitive, inflexible and sedentary nature, which is incompatible with the neurocognitive needs of many children, including those with ADHD [47]. The findings from this study, along with previous work [9,13,14,19], underscore the need for classrooms to offer opportunities for more movement and full body engagement, to not only evoke the necessary neural processes needed for effective executive functioning, but to make the learning space more engaging for those where monotony is more harmful for motivation and attention. Perhaps stimulant medication would be less necessary if the environments in which we expect children to operate considered their underlying neural machinery and holistic bodily needs. This work also lends support to families looking for alternative treatments beyond pharmaceuticals to support their child’s ADHD symptoms.

### 4.2. Limitations

The present study is subject to several limitations. The first is the reliance on a single executive functioning task (Stroop), which may limit the generalizability of the findings; a more robust estimate of executive functioning would be provided if multiple measures were used to assess inhibitory control. Future research may consider employing the Attention Network Task (ANT), which provides insight into the complex mechanisms of attention by distinguishing three components: altering, orienting, and executive control [48,49]. Indeed, the ANT has been applied in previous studies examining ADHD in children and may be particularly well-suited for evaluating the effects of movement-based interventions in ADHD [48,49]. A further limitation is the lack of screening for psychiatric disorders beyond the exclusion criteria, with no control for common comorbidities, including oppositional defiant disorder (ODD), mood disorders, and anxiety disorders. As these disorders are highly comorbid with ADHD, recruiting participants with ADHD diagnoses exclusively would be extremely difficult, if not unfeasible. Additionally, the experimental design of the study limited analyses to a single-subject level, with statistical software unable to support a typical second-level GLM based on the design. Reporting group-level significance would strengthen the statistical interpretation of the current data and reduce perceptions of selective reporting. Further, individual difference factors such as symptom severity or subtype were not controlled for in the current study; future work would stand to benefit from matching children on levels of severity and subtype to unpack whether these are important moderating factors in the relationship between movement and neurocognitive functioning in ADHD. And lastly, desk cycling was self-paced, with cycling intensity varying slightly across children. Cycling intensity was intentionally left uncontrolled to allow participants to adjust the speed based on their own internal sense of stimulation needs.

## 5. Conclusions

This study suggests that for children with ADHD who cannot tolerate psychostimulant medication due to adverse side effects, incorporating movement into attention-demanding activities may offer comparable benefits by supporting focused attention and cognitive performance. It is essential that environments like classrooms and workplaces offer outlets for stimulating engagement of the body and mind to create the necessary conditions for focused attention to emerge so that children can thrive.

## Figures and Tables

**Table 1:** Demographic data in the ADHD medicated group, ADHD unmedicated group, and Control group.

Characteristic	ADHD Medicated (*n* = 15)	ADHD Unmedicated (*n* = 11)	Control (*n* = 24)
Child’s Age (years), mean (SD)	9.73 (1.53)	10.09 (1.76)	10.21 (1.06)
Guardian’s Age (years), mean (SD)	44.2 (7.17)	38.82 (8.20)	45.17 (4.62)
Participant’s Sex			
Male	7	9	14
Female	8	2	8
Guardian’s Sex			
Male	2	3	4
Female	13	8	20
Guardian’s Education Level (*n*)			
Some high school, no diploma	0	0	0
High school graduate, diploma or the equivalent	0	1	0
Some college credit, no degree	1	0	2
Trade/technical/vocational training	3	0	1
Associate degree	1	2	1
Bachelor’s degree	6	3	8
Master’s degree	2	3	7
Professional degree	3	1	5
No response	0	1	0
Guardian’s Employment (*n*)			
Employed for wages	10	7	13
Self-employed	3	2	6
Unemployed	1	1	3
Student	0	1	0
Household Income			
Prefer not to say	1	1	4
<$30,000	0	1	1
$30,000–$40,000	0	0	1
$40,000–$50,000	0	1	1
$50,000–$60,000	1	2	3
$60,000–$70,000	2	0	1
$70,000–$80,000	0	0	1
$80,000–$90,000	2	0	2
$90,000–$100,000	3	1	0
>$100,000	6	5	8
Age of ADHD diagnosis (*n*)			
Unsure	0	3	-
3–5	2	2	-
6–8	9	4	-
9–12	3	2	-
ADHD Subtype (*n*)			
Predominantly inattentive	3	2	-
Predominantly hyperactive	2	4	-
Combined subtype	11	5	-
Unsure/no diagnosis given			-
ADHD Severity (*n*)			
Low	3	5	-
Moderate	6	3	-
High	6	3	-
Number of ADHD Medications			
1	13	3	-
2	2	0	-
Type of ADHD Medication			
Methylphenidate Stimulants (i.e., Concerta, Biphentin, Methylphenidate HCI)	11	2	-
Amphetamine Stimulants (i.e., Vyvanse)	3	1	-
Non-Stimulants (i.e., Strattera, Intuniv)	3	0	-
None	0	8	-
Length of ADHD Medication			
<6 months	1	2	-
6–11 months	5	1	-
1–3 years	6	1	-
3+ years	2	0	-
Comorbid Diagnosis			
Yes	8	0	0
No	7	11	24
Medicated for Comorbid Diagnosis			
Yes	4	0	0
No	11	11	24

**Table 2:** Descriptive statistics for Stroop outcome variables by group and condition.

	Unmedicated ADHD (*n* = 11)		Medicated ADHD (*n* = 14)		Controls (*n* = 24)	
	Stationary M (SD)	Movement M (SD)	Stationary M (SD)	Movement M (SD)	Stationary M (SD)	Movement M (SD)
**Total RT**	1144 (809)	1003 (247)	1252 (502)	1083 (404)	1103 (420)	968 (200)
**Congruent RT**	1265 (1264)	935 (234)	1241 (618)	1040 (394)	1054 (447)	932 (248)
**Incongruent RT**	1068 (524)	1071 (371)	1272 (469)	1134 (466)	1131 (475)	1007 (233)
**Total PC**	0.87 (0.13)	0.95 (0.07)	0.95 (0.06)	0.91 (0.11)	0.90 (0.13)	0.92 (0.11)
**Congruent PC**	0.95 (0.15)	0.97 (0.09)	0.96 (0.09)	0.90 (0.16)	0.96 (0.12)	0.93 (0.12)
**Incongruent PC**	0.80 (0.18)	0.93 (0.11)	0.93 (0.11)	0.92 (0.13)	0.84 (0.23)	0.89 (0.17)

## Data Availability

The data presented in this study are available on request from the corresponding author due to ethical restrictions.

## Supplementary Materials

The following supporting information can be downloaded at https://www.mdpi.com/article/10.3390/brainsci15101107/s1. Table S1: fNIRS general linear model (GLM) contrast results by group.
