# Supplementary material for: Medication vs. Movement in ADHD: Interaction Between Medication and Physical Activity on Neurocognitive Functioning
Source: Brain Sci. 2025 Oct 15;15(10):1107. doi: 10.3390/brainsci15101107 (PMC12562749; doi:10.3390/brainsci15101107)
Supplement: Supplementary file 1 [file brainsci-15-01107-s001.zip › brainsci-3895887-supplementary.pdf]

Table S1. fNIRS general linear model (GLM) contrast results by group.

Group	Participant ID	DLPFC ROI	HbO		HbR	
			T-value	P-value	T-value	P-value
		F1	-4.93	<.001	11.32	<.001
**Unmedicated**	2	F3	5.45	<.001	-15.14	<.001
		F5	10.36	<.001	-9.06	<.001
		F1	-4.24	<.001	-11.52	<.001
**Unmedicated**	17	F3	-6.65	<.001	-8.12	<.001
		F5	7.68	<.001	-12.82	<.001
		F1	-5.28	<.001	-0.61	.53
**Unmedicated**	32	F3	-2.86	.004	-17.54	<.001
		F5	-0.89	.38	-6.04	<.001
		F1	-11.84	<.001	11.66	<.001
**Unmedicated**	9	F3	-13.27	<.001	12.31	<.001
		F5	-7.3	<.001	-.15	.88
		F1	2.02	.04	10.87	<.001
**Unmedicated**	36	F3	24.42	<.001	-1.89	.05
		F5	-10.14	<.001	-0.76	.44
		F1	9.51	<.001	-5.8	<.001
**Unmedicated**	37	F3	2.23	.02	-26.33	<.001
		F5	12.74	<.001	-26.08	<.001
		F1	-1.91	.05	-14.27	<.001
**Unmedicated**	39	F3	-0.07	.94	-8.89	<.001
		F5	0.85	.39	4.95	<.001
		F1	-0.55	.57	-11.11	<.001
**Unmedicated**	29	F3	8.16	<.001	-19.78	<.001
		F5	-10.57	<.001	-1.38	.16
		F1	14.62	<.001	-14.01	<.001
**Unmedicated**	35	F3	2.12	.03	4.93	<.001
		F5	20.55	<.001	-17.88	<.001
		F1	-11.11	<.001	-1.64	.1
**Unmedicated**	47	F3	-1.97	.05	7.94	<.001
		F5	-6.46	<.001	16.41	<.001
		F1	-5.21	<.001	19.25	<.001
**Unmedicated**	51	F3	-10.77	<.001	33.08	<.001
		F5	-1.41	.16	-7.54	<.001
		F1	-21.78	<.001	-1.86	.06
**Medicated**	3	F3	5.4	<.001	-.25	.80
		F5	-13.09	<.001	-11.33	<.001
		F1	12.71	<.001	-5.75	<.001
**Medicated**	13	F3	18.36	<.001	3.16	.002
		F5	25.12	<.001	-7.6	<.001
		F1	-1.5	.12	32	<.001
**Medicated**	27	F3	.86	.38	16	<.001

		F5	-3.25	.001	-3.8	<.001
		F1	1.19	.23	-1.09	.27
**Medicated**	24	F3	6.61	<.001	4.96	<.001
		F5	5.71	<.001	16	<.001
		F1	-2.09	.03	5.43	<.001
**Medicated**	28	F3	-8.65	<.001	0.57	.56
		F5	-24.6	<.001	1.92	.05
		F1	-23.92	<.001	9.63	<.001
**Medicated**	53	F3	-9.21	<.001	-5.23	<.001
		F5	-17.1	<.001	2.78	.01
		F1	-0.51	.6	-1.53	.12
**Medicated**	30	F3	-1.99	.05	12.81	<.001
		F5	-10.72	<.001	16.34	<.001
		F1	-5.36	<.001	1.14	.25
**Medicated**	31	F3	-7.27	<.001	-8.08	<.001
		F5	10.08	<.001	-9.27	<.001
		F1	-4.65	<.001	30.04	<.001
**Medicated**	41	F3	-10.6	<.001	26.55	<.001
		F5	-18.7	<.001	0.35	.71
		F1	14.63	<.001	4.09	<.001
**Medicated**	40	F3	7.76	<.001	2.53	.01
		F5	-10.16	<.001	12.52	<.001
		F1	-8.23	<.001	-7.21	<.001
**Medicated**	42	F3	-3.78	<.001	16.59	<.001
		F5	-10.64	<.001	-4.74	<.001
		F1	-23.92	<.001	9.63	<.001
**Medicated**	53	F3	-9.21	<.001	-5.23	<.001
		F5	-17.1	<.001	2.78	.01
		F1	17.01	<.001	11.12	<.001
**Medicated**	14	F3	12.76	<.001	14.41	<.001
		F5	-7.14	<.001	9.21	<.001
		F1	-9.21	<.001	8.63	<.001
**Medicated**	52	F3	8.91	<.001	16.03	<.001
		F5	-6.53	<.001	5.47	<.001
		F1	-8.2	<.001	-13.21	<.001
**Medicated**	49	F3	15.96	<.001	17.9	<.001
		F5	-6.29	<.001	3.5	.001
		F1	9.31	<.001	13.93	<.001
**Medicated**	5	F3	9.84	<.001	-.12	.90
		F5	6.01	<.001	1.61	.11
		F1	4	<.001	-7	<.001
Control	4	F3	14	<.001	-8	<.001
		F5	4	<.001	9	<.001
		F1	-3	.004	2.4	.01
Control	11	F3	-13	<.001	1.5	.11
		F5	7	<.001	12	<.001
		F1	14	<.001	-9	.06
Control	15	F3	-12	<.001	17	.80

		F5	9	<.001	-15	<.001
		F1	5.4	<.001	-.3	.77
Control	18	F3	-1.6	.11	12	.90
		F5	15	<.001	9	<.001
		F1	-10	<.001	17	<.001
Control	20	F3	-19	<.001	35	<.001
		F5	-1.6	.1	7.1	<.001
		F1	-2.5	.01	16	<.001
Control	7	F3	-4	<.001	-2.5	.01
		F5	-16	<.001	11	<.001
		F1	2.16	.03	5.7	<.001
Control	10	F3	-17	<.001	2.2	.02
		F5	5.3	<.001	.5	.59
		F1	-25	<.001	14	<.001
Control	16	F3	-12	<.001	-8	<.001
		F5	-24	<.001	-13	<.001
		F1	-27	<.001	19	<.001
Control	23	F3	-2.7	.01	12	<.001
		F5	-15	<.001	10	<.001
		F1	4.5	<.001	2.9	.004
Control	22	F3	9.1	<.001	-12.7	<.001
		F5	4.2	<.001	3.7	<.001
		F1	-4.9	<.001	-11.3	<.001
Control	6	F3	-1.7	.09	4.3	<.001
		F5	2.4	.02	-5.7	<.001
		F1	9.1	<.001	3.7	<.001
Control	8	F3	6.4	<.001	-18.2	<.001
		F5	20.1	<.001	-5.8	<.001
		F1	3.5	<.001	.8	.4
Control	12	F3	-2.1	.03	-.7	.5
		F5	3.9	<.001	-26.9	<.001
		F1	16.9	<.001	12.09	<.001
Control	25	F3	5.4	<.001	-9.2	<.001
		F5	-4.7	<.001	12.2	<.001
		F1	-7	<.001	-12	<.001
Control	26	F3	-14.7	<.001	-6.9	<.001
		F5	-13.1	<.001	-3	<.001
		F1	-13.47	<.001	-25.72	<.001
Control	33	F3	-30.3	<.001	-30.66	<.001
		F5	-4.01	<.001	-27.2	<.001
		F1	2.99	.002	0.19	.84
Control	34	F3	7.52	<.001	10.62	<.001
		F5	-6.84	<.001	27.21	<.001
		F1	12.67	<.001	8.51	<.001
Control	38	F3	12.77	<.001	2.25	.02
		F5	-13.75	<.001	19.94	<.001
		F1	-2.06	.04	-16.97	<.001
Control	43	F3	8.53	<.001	-6.67	<.001

		F5	18.24	<.001	3.53	<.001
Control	48	F1	-6.85	<.001	-5.41	<.001
		F3	10.78	<.001	12.01	<.001
		F5	6.69	<.001	-3.46	<.001
Control	50	F1	10.35	<.001	6.48	<.001
		F3	15.56	<.001	8.23	<.001
		F5	27.14	<.001	19.19	<.001
Control	54	F1	-3.34	<.001	11.26	<.001
		F3	-10.55	<.001	33.64	<.001
		F5	-8.76	<.001	25.88	<.001
Control	55	F1	-13.38	<.001	-27.09	<.001
		F3	15.75	<.001	5.64	<.001
		F5	28.9	<.001	-13.58	<.001

Note. HbO (oxygenated hemoglobin), HbR (deoxygenated hemoglobin), DLFPC (dorsolateral prefrontal cortex), ROI (region of interest), F1 (fNIRS channel source 4-detector 2), F3 (fNIRS channel source 1-detector 2), F5 (fNIRS channel source 1-detector 1).
